# TGF-b Superfamily Cytokine MIC-1/GDF15 Is a Physiological Appetite and Body Weight Regulator

**DOI:** 10.1371/journal.pone.0055174

**Published:** 2013-02-28

**Authors:** Vicky Wang-Wei Tsai, Laurence Macia, Heiko Johnen, Tamara Kuffner, Rakesh Manadhar, Sebastian Beck Jørgensen, Ka Ki Michelle Lee-Ng, Hong Ping Zhang, Liyun Wu, Christopher Peter Marquis, Lele Jiang, Yasmin Husaini, Shu Lin, Herbert Herzog, David A. Brown, Amanda Sainsbury, Samuel N. Breit

**Affiliations:** 1 St Vincent’s Centre for Applied Medical Research, St Vincent’s Hospital and University of New South Wales, Sydney, New South Wales, Australia; 2 Neuroscience Program, Garvan Institute of Medical Research, Sydney, New South Wales, Australia; 3 Diabetes Research Unit, Novo Nordisk A/S, Maaloev, Denmark; 4 School of Biotechnology and Biomolecular Sciences, University of New South Wales, Sydney, New South Wales, Australia; Pennington Biomedical Research Center, United States of America

## Abstract

The TGF-b superfamily cytokine MIC-1/GDF15 circulates in all humans and when overproduced in cancer leads to anorexia/cachexia, by direct action on brain feeding centres. In these studies we have examined the role of physiologically relevant levels of MIC-1/GDF15 in the regulation of appetite, body weight and basal metabolic rate. MIC-1/GDF15 gene knockout mice (MIC-1^−/−^) weighed more and had increased adiposity, which was associated with increased spontaneous food intake. Female MIC-1^−/−^ mice exhibited some additional alterations in reduced basal energy expenditure and physical activity, possibly owing to the associated decrease in total lean mass. Further, infusion of human recombinant MIC-1/GDF15 sufficient to raise serum levels in MIC-1^−/−^ mice to within the normal human range reduced body weight and food intake. Taken together, our findings suggest that MIC-1/GDF15 is involved in the physiological regulation of appetite and energy storage.

## Introduction

The regulation of food intake, energy storage and energy expenditure are tightly controlled by complex homeostatic mechanisms. These involve conveying information about total body nutritional and energy status, as well as the presence of nutrients in the gut lumen and the circulation, to the central nervous system (CNS), notably via the release of hormones from the gut and adipose tissue. The CNS, in turn, initiates the transcription and release of neuropeptides in the hypothalamus and brainstem, which then modulate feeding and metabolism in order to maintain energy homeostasis [1]–[3]. Gut and adipose tissue-derived hormones may increase or decrease appetite. Satiety inducing gut hormones are released in response to food intake, and mediate rapid regulation of appetite [4], whilst insulin from the pancreas, and leptin, the circulating levels of which increase with increasing fat mass, are involved in long-term regulation of energy balance [5]. In disease states, other regulatory molecules have also been identified that may have an important role in regulating energy homeostasis. One such molecule is MIC-1/GDF15 [6], an unusual and divergent member of the TGF-b superfamily, sometimes known as GDF15, PLAB, NAG-1 or PTGFB [6]–[8]. We have previously demonstrated that this cytokine is an important cause of the anorexia/cachexia of cancer [9].

Many patients with different types of cancers have elevated circulating levels of MIC-1/GDF15 [10]–[13]. Serum MIC-1/GDF15 levels can rise dramatically in advanced cancer, from the normal mean of about 450 pg/ml to up to 10,000–100,000 pg/ml [8], [10]. MIC-1/GDF15 levels of above 5,000–8,000 pg/ml cause severe anorexia/cachexia [9], and animal studies demonstrate that this is likely due to direct actions of MIC-1/GDF15 on feeding centres in the brainstem and hypothalamus [9]. In addition, elevated serum MIC-1/GDF15 levels have also been linked to cachexia associated with chronic renal [9] and cardiac failure [14], [15]. Lastly, as we have previously reported, a mouse model with transgenic overexpression of MIC-1/GDF15 also displayed decreased body weight and fat mass, in association with a decrease in food intake [16]. While these data demonstrated a clear causal link between markedly elevated MIC-1/GDF15 serum levels and MIC-1/GDF15 in mediating changes in energy intake and storage leading to cachectic syndromes, the role of physiological circulating levels of MIC-1/GDF15 in energy homeostasis is unknown.

To start addressing the biological actions of physiological concentrations of MIC-1, we compared body composition and food intake between MIC-1/GDF15 deficient (MIC-1^−/−^) mice and syngeneic wildtype (MIC-1^+/+^) mice. We also analysed possible differences in metabolic activity by comparing respiratory exchange ratio, energy expenditure and physical activity between genotypes. Lastly, we infused MIC-1^−/−^ and MIC-1^+/+^mice with human MIC-1/GDF15 to increase circulating MIC-1/GDF15 concentrations to various levels within the physiological range in order to evaluate the effects on body weight and appetite. These studies demonstrate that MIC-1/GDF15 is likely to play a role in the physiological regulation of energy intake and expenditure.

## Materials and Methods

All procedures were approved and performed in accordance with the guidelines of the Garvan Institute and St. Vincent’s Hospital Animal Experimentation Ethics Committee (AEC 11/36). All animals were maintained under a controlled temperature of 22°C and a 12-h dark and 12-h light cycle. Mice were given *ad libitum* access to standard rodent chow (Gordon’s Specialty Stock Feeds, Yanderra, NSW, Australia) and water.

### Generation of MIC-1^−/−^ Mice

Mice with germline-deleted MIC-1/GDF15 (MIC-1^−/−^) was generated by Ozgene (Ozgene Pty Ltd., Bentley DC, WA Australia). These mice have a complete deletion of the second of two exons of the MIC-1/GDF15 gene. This effectively deleted the poly A tract and amino acids 94–302 of MIC-1/GDF15, including all of the mature bioactive domain and most of the propeptide region. The founder mice were bred for more than 10 generations onto a C57BL/6 background.

### MIC-1/GDF15 Reagents

All MIC-1/GDF15 antibodies and recombinant protein were prepared as previously described [17]. Briefly, recombinant human MIC-1/GDF15 was expressed and purified to homogeneity from conditioned medium of the yeast *Pichia pastoris* that is free from LPS. The monoclonal antibody against human MIC1/GDF15 (mAb-26) was purified by protein G affinity chromatography.

### Indirect Calorimetry

Indirect calorimetry was performed in age matched mice at 12–16 weeks of age using an eight-chamber open-circuit calorimeter (Oxymax Series; Columbus Instruments, Columbus, OH, USA). Mice were weighed and singly housed in Plexiglass cages (20.1×10.1×12.7 cm) and were left to acclimatized for 24 h before commencement of 48 h-recordings. Oxygen consumption (Vo_2_) and carbon dioxide (Vco_2_) were measured every 15 min. The respiratory exchange ratio (RER) was calculated as the quotient of Vco_2_/Vo_2_, with an RER of 1 indicating 100% carbohydrate oxidation and an RER of 0.7 indicating 100% fat oxidation [18]. Energy expenditure was measured as production of kcal of heat and was calculated as Calorific Value (CV) × Vo_2_, where CV is 3.815+1.232 × RER [19]. Data for the 24-h monitoring period was averaged for 1-h intervals for RER and energy expenditure (kcal/h). Ambulatory activity was recorded with an OPTO-M3 infrared beam sensor system (Columbus Instruments, Columbus, OH). The senor beams were aligned on both x and y-axes directions. Data was collected at 1 min intervals at the same time as the indirect calorimetry measurements. The recording of ambulatory activity (locomotion) only counts the broken beam when a consecutive adjacent beam is broken, and does not include the same beam being broken repeatedly [20]. The total counts of x and y-axes for every 1-h interval from individual mouse were used for analysis of ambulatory activity.

### Measurement of Body Composition

Whole body fat mass and lean mass were measured in MIC-1^−/−^ and control mice at 12–14 weeks of age. Animals were subjected to dual-energy X-ray absorptiometry (DXA; PIXImus2 mouse densitometer; GE Health-care, Waukesha, WI) after anesthetized with isoflurane. The head and the tail were excluded from all the measurements.

### Tissue Collection

Upon completion of metabolic and body composition measurements, mice at 14–16 weeks of age were sacrificed by cervical dislocation. Muscles (gastrocnemius and tibialis), whole interscapular brown adipose tissue, as well as white adipose tissue depots (inguinal, epididymal, mesenteric and retroperitoneal) were collected and weighed. Total white adipose tissue (WATt) mass is defined as the sum of the mass of these four WAT depots.

### Subcutaneous Osmotic Pump Implantation

Recombinant human MIC-1/GDF15 was reconstituted in 4 mM HCl and loaded into a 7-day-Mini-osmotic pump (model 1007D, ALZET Osmotic pump, Cupertino, CA) to deliver 1 ug/24 h/20 gBW at delivery rate of 0.5 ul/h. MIC-1/GDF15 or vehicle-loaded pumps were implanted subcutaneously in the interscapular region of 10–14 week-old MIC-1^−/−^ or MIC-1^+/+^ mice. Briefly, animals were anesthetized by inhalation of isoflurane then shaved and disinfected over the implantation site. A small incision was made across the midline and slightly posterior to the scapula, then a hemostat was used for blunt dissection into the subcutaneous space to create a space for the pump, which was inserted with the delivery portal oriented caudally. The wound was closed with two 9 mm wound clips. Mice were sacrificed at either day 5 or 6 after implantation for blood collection.

### Body Weight and Food Intake Study

Mice were transferred from group house on soft bedding to individual cages with paper-towel and allowed to acclimatize for three days before commencement of food intake measurements. Body weight, food spillage on the bedding and food in the food hopper were weighed at between 9∶00 and 10∶00 h. Food consumption was calculated using the weight of food pellets left in the hopper as well as the weight of food (minus feces) spilled on the bedding [9].

### Quantification of Serum MIC-1/GDF15 Levels in Mice

Mice were sacrificed at day 6 after implantation of osmotic minipump. Blood samples were collected by cardiac puncture and allowed to clot by standing for 2 hour at 4°C. After centrifugation, the collected sera were stored at −70°C for assay of human MIC-1/GDF15 by a previously described, in house ELISA [21], [22].

### Statistical Analysis

The difference in body weight between the MIC-1^−/−^ and MIC-1^+/+^ animal over 52 weeks were average of 12 animals in each group for each age. Comparison of energy expenditure between genotypes were performed first by determining the equality of variance between the group with Levene’s test followed by ANCOVA with lean body mass as covariate, which also tested the homogeneity of regression line slopes between the groups (SPSS version 20, Chicago, IL). All data for energy expenditure were readjusted at a common lean mass generated by ANOVA and subsequent comparison between the groups was performed using repeated-measure ANOVA or t-test. RER and physical activity collected over 24-h were averaged for the whole 24-h period, as well as for the light and dark phase, comparison between genotypes within the same sex or between difference treatments were performed using an repeated measures ANOVA and unpaired-two-tailed t-test or with when values over different times were analyzed. These analyses were performed using GraphPad Prism (PRISM 4, GraphPad, San Diago, CA). Equation of Power analysis was used for determining animal number required for 95% power and 5% significance in food intake between male genotypes, which was derived by using the standard deviation and difference between mean in food intake of genotypes [23].

## Results

### MIC-1^−/−^ Mice have Increased Body Weight and Fat Mass

**Figure 1:**
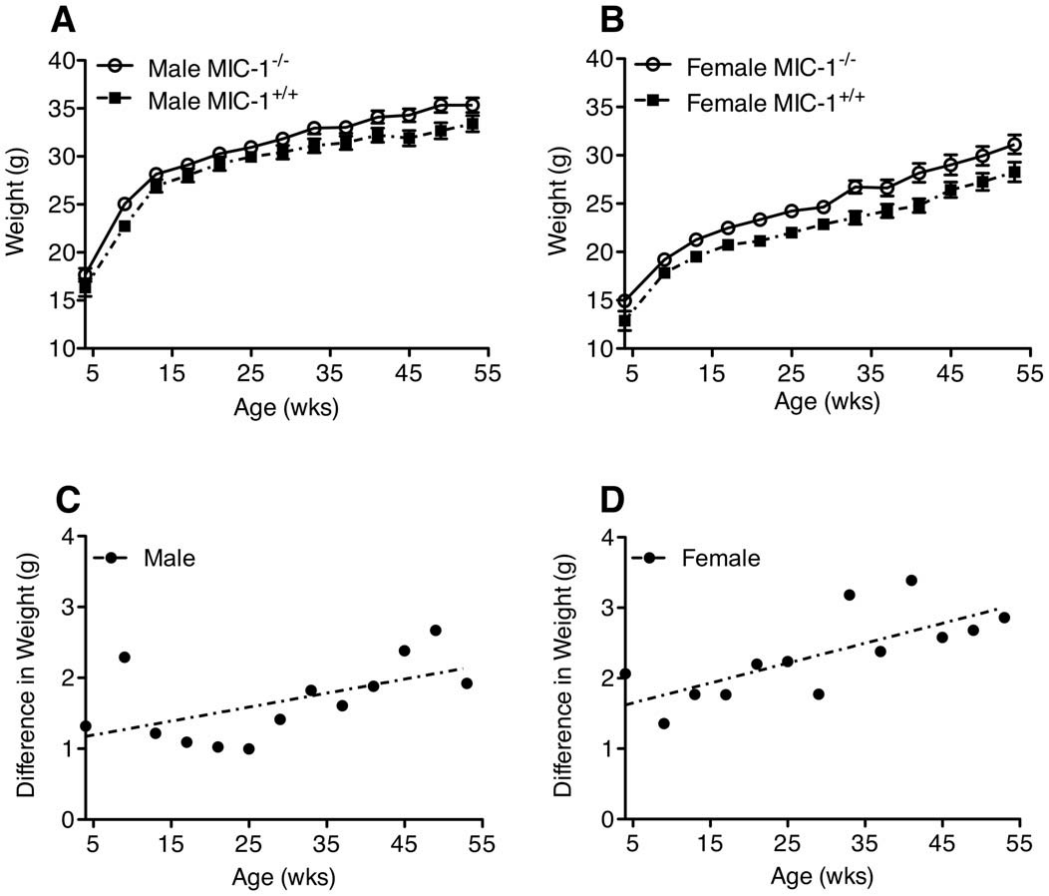
MIC-1^−/−^ are heavier than MIC-1^+/+^ mice. (A) Male and (B) female MIC-1^−/−^ mice and syngeneic control MIC-1^+/+^ mice were weighed once every four weeks from age of 4 weeks to 1 year. Both male and female MIC-1^−/−^ mice were on average 6–10% heavier than the MIC-1^+/+^ mice (male *n* = 13/group, *p* = 0.04; female *n* = 13/group, *p* = 0.01 *repeated measures ANOVA*). The weight difference between genotypes appeared from the age of 4 weeks with increased weight differences with ageing in both (C) male and (D) female mice (male *n* = 13/group, *p* = 0.044, *r^2^* = 0.32; female *n* = 13/group *p*<0.001, *r^2^* = 0.55, *linear regression*). Data expressed as mean ± SE.

To investigate whether MIC-1/GDF15 might contribute to the physiological regulation of energy homeostasis, the body weight of MIC-1^−/−^ mice and syngeneic controls were monitored between the ages of 4 weeks to 1 year. Male MIC-1^−/−^ mice were on average 6±0.6% heavier than male MIC-1^+/+^ control mice of the same age, and female MIC-1^−/−^ mice weighed on average 10±0.7% more than female controls (Fig. 1A and 1B, male *p* = 0.04; female *p* = 0.01). For both male and female mice, the weight differences between genotypes increased significantly over time (Fig. 1C and 1D, female *p*<0.01; male *p* = 0.04).

**Figure 2:**
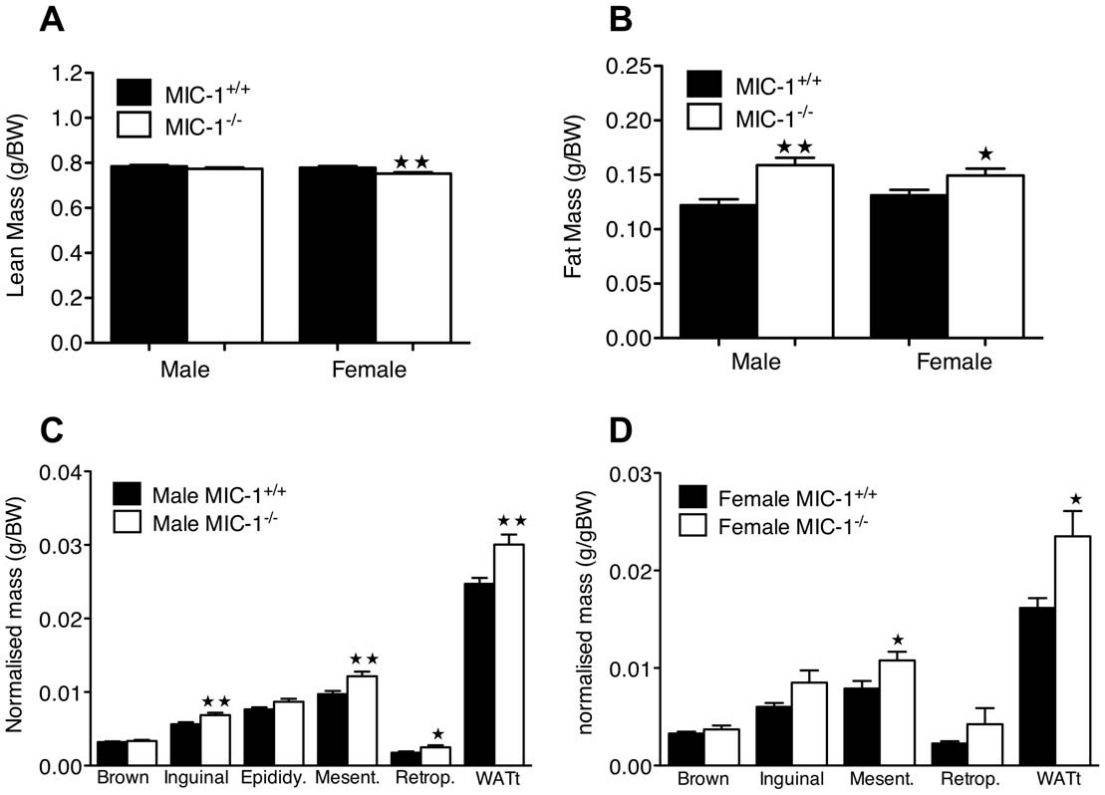
**Lack of MIC-1 signaling alters the regulation of body fat depots.** (A) Whole body lean mass and (B) fat mass was determined by dual energy X-ray absorptiometry (DXA) in 15 mice per group at 12–14 weeks of age. Female MIC-1^−/−^ mice had lower lean mass relative to control mice (*p<*0.01, *n = *15/group, *t-test*), Both male and female MIC-1^−/−^ mice had significantly higher fat depot mases compared to synergic control (male *p<*0.01, female *p = *0.04, *n = *15/group, *t-test*). Mass of individual white adipose tissue depots were measured in (C) male and (D) female mice (*n = *9/group) aged between 14–16 weeks. Fat masses, namely inguinal, epididymal (Epididy), mesenteric (Mesent), retroperitoneal (Retrop), and total white adipose tissue (WATt) were normalized to body weight. In both male and female MIC-1^−/−^ mice, WATt depots were significantly higher than the synergic control (male *p<*0.01, female *p = *0.02, *n = *9/group, *t-test*). Data are means ± SE. Significance indicated as (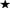) for *p<*0.05 or (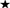 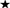) for *p<*0.01.

To understand what might have contributed to the increased body weight in MIC-1^−/−^ mice, whole body lean and fat mass were determined by dual energy X-ray absorptiometry (DXA). Whilst male MIC-1^−/−^ mice show a similar lean body mass to age and sex matched controls, female MIC-1^−/−^ mice had a significantly lower lean body mass than age and sex matched MIC-1^+/+^ mice (Fig. 2A, male *p* = 0.21; female *p*<0.01). Both male and female MIC-1^−/−^ mice had a significantly higher total fat mass relative to the sex matched MIC-1^+/+^ mice (Fig. 2B, male *p*<0.01; female *p* = 0.04).

To further delineate differences in body composition, tissue specific lean mass and fat depot weights were measured directly. There was no significant difference between MIC-1^+/+^ and MIC-1^−/−^ mice of either sex in the relative mass of either the gastrocnemius or tibialis muscles (data not shown), and no differences were observed in mass of brown adipose tissue (Fig. 2C, 2D, male *p* = 0.35, female *p* = 0.35). However, there was a significant increase in total white adipose tissue mass (WATt), normalized to body weight, in both male and female MIC-1^−/−^ animals (Fig. 2C, 2D, male *p<*0.01, female *p = *0.02). This was associated with marked significant increases in the weight of the inguinal, mesenteric and/or retroperitoneal fat depots in MIC-1^−/−^ compared to control mice (Fig. 2C, 2D). These data indicate that MIC-1/GDF15 plays a role in regulating body composition and energy storage in mice.

### Female but not Male MIC-1^−/−^ Mice have Increased Spontaneous Food Intake

**Figure 3:**
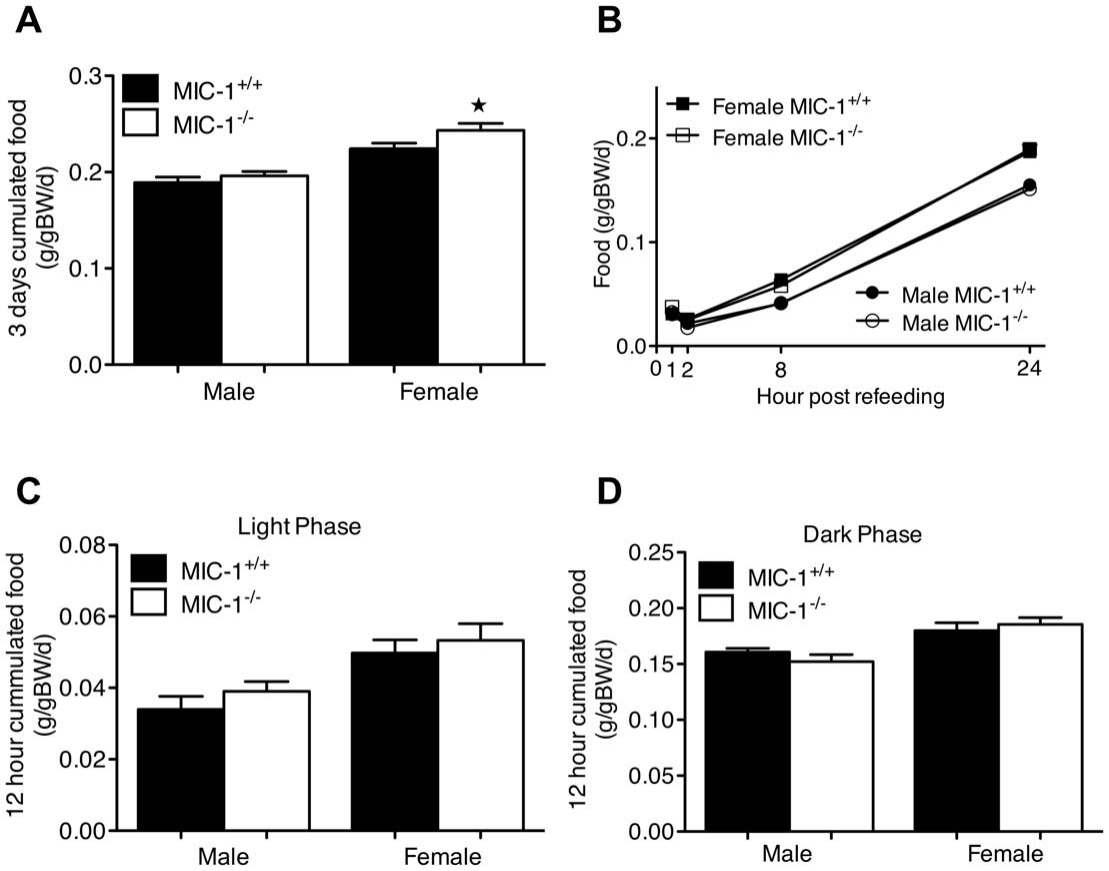
**Female MIC-1^−/−^ mice eat more.** (A) Spontaneous 3 day cumulated food intake was measured in male and female MIC-1^−/−^ and control mice at 13 weeks of age. All mice were fed with standard chow diet ad libitum. Similar food intake was observed between male genotypes (*p = *0.3, *n = *8/group, t-test), female MIC-1^−/−^ mice had higher food intake relatively to the control mice (*p = *0.05, *n = *8/group). (B) Cumulated 24-hour fasting-induced food intake of was performed with the same group of mice at age of 14 weeks. MIC-1^−/−^ and control mice were fasted for 24 hours before re-introduction of food and spillage were collected at indicated time points, no genotypic difference were observed both male and female mice. Food intake at (C) light and (D) dark phase was also measured in the same group of mice at age of 12 weeks. No significant changes were observed between MIC-1^−/−^ and control mice in both sexes. Data are normalized to body weight plotted as means ± SE. Significance indicated as (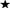) for *p≤*0.05.

To examine possible causes for the increased body weight and fat mass in the MIC-1^−/−^ mice, we first studied their spontaneous food intake. Female but not male MIC-1^−/−^ had significant increased food intake compared to the age and sex-matched control mice, both in absolute terms (15.59±0.67 versus 12.77±0.88 g/gBW/d in female knockout and control mice, respectively) and when normalized to body weight (p = 0.05 for female mice (Fig. 3A). This data suggested that the increased body weight in female MIC-1^−/−^ is at least partly due to increased food intake. Whilst the 3.7% difference in food intake between male MIC-1^−/−^ and MIC-1^+/+^ was not statistically significant, this may reflect the capacity of our method to detect small differences in food intake. Power analysis indicates that to determine with 95% certainty whether this 3.7% difference in food intake was significant would require 126 mice of each genotype. As, over a more prolonged period, a difference in 3 days-accumulated food intake of as little as 3.7% is likely to be able alter body weight and composition [23], in this study, we cannot exclude such a small difference being present.

As the timing of food intake can influence energy storage independently of total intake [24], we also measured food intake after fasting, as well as during the light and dark phases in all animals (Figs 3B, 3C, 3D). However, there was no difference between knockout and control mice of either sex with respect to re-feeding after a 24-hour fast (Fig. 3B, *p = *0.8 for both sexes). Additionally, there were no significant differences in the pattern of food intake in the light and dark phase between male and female MIC-1^−/−^ and control mice (Fig. 3C, 3D).

### Female but not Male MIC-1^−/−^ Mice have Lower Total Energy Expenditure

**Figure 4:**
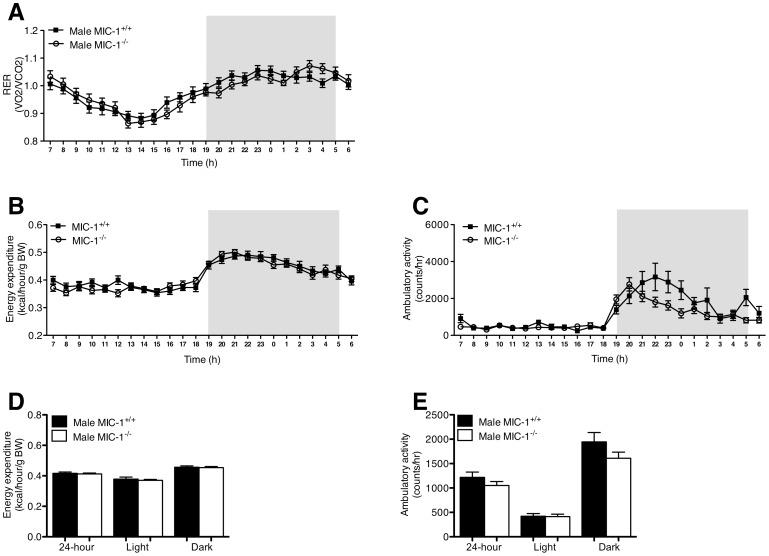
**Male MIC^−/−^ mice exhibit similar metabolic activity to their synergic control mice.** Metabolic activity of male MIC-1^−/−^ and control mice with groups of 16 at age between 14–16 weeks was determined by time course of (A) respiratory exchange rate (RER), (B) energy expenditure and (C) ambulatory activity. Energy expenditure was adjusted for lean mass via ANCOVA (common lean mass = 25.65 g). (D) Energy expenditure and (E) ambulatory activity were also presented as total for 24 hour, light phase and dark phase. Data are normalized to body weight and plotted as means ± SE.

**Figure 5:**
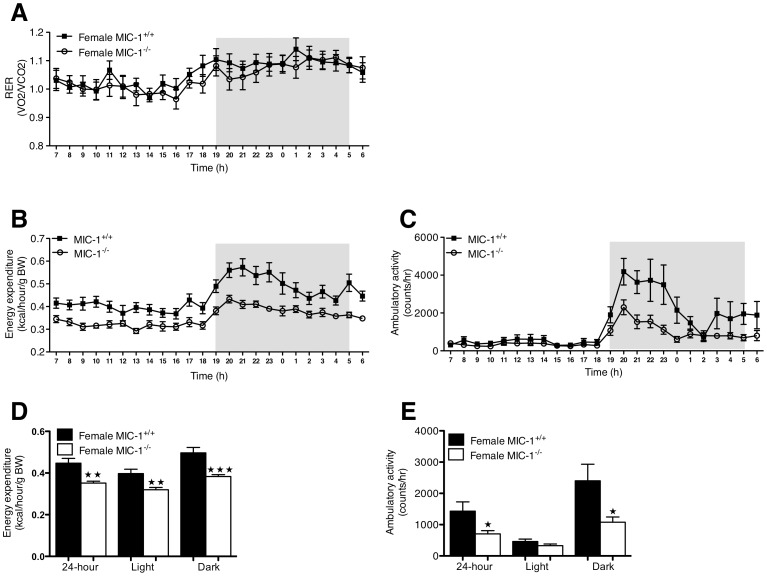
**Female MIC^−/−^ mice exhibit lower metabolic activity than their synergic controls.** Metabolic activity of female MIC-1^−/−^ and control mice with groups of 9 at age between 14–16 weeks was determined by time course of (A) respiratory exchange rate (RER), (B) energy expenditure and (C) ambulatory activity. Energy expenditure (EE) was adjusted for lean mass via ANCOVA (common lean mass = 18.72 g), EE were significantly lower measured over 24 hour in MIC-1^−/−^ mice (*p = *0.001, *n = *9/group, *repeated measures ANOVA*). (D) MIC-1^−/−^ also displayed lower total EE in time courses over 24 hour, light phase and dark phase (*p = *0.001. *p = *0.005 and *p<*0.001, respectively, *n = *9/group, t-test). (E) Physical activity in dark phase were significantly lower in MIC-1^−/−^ mice (*p = *0.03, *n = *9, t-test). Data are normalized to body weight and plotted as means ± SE. Significance indicated as (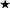) for *p<*0.05 or (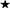 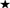) for *p<*0.01, or (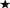 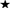 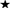) for *p<*0.001.

To further investigate possible mechanisms underlying the increases in body weight and adiposity of male and female MIC-1^−/−^ versus MIC-1^+/+^ mice, we compared their respiratory exchange ratio (RER), energy expenditure and physical activity (Figs 4 and 5). The increased body weight and adiposity of MIC-1^−/−^ animals does not appear to result from differential use of lipids versus carbohydrate as oxidative fuel sources as there was no difference in RER between genotypes (Fig. 4A, 5A). Female mice, MIC-1^−/−^ animals exhibit significantly lower energy expenditure normalized to bodyweight compared to the age matched control MIC-1^+/+^ mice (*p<*0.01, Fig. 5B, 5D). This difference may be partially attributed to a decrease in physical activity, since physical activity was significantly decreased during the dark phase in female MIC-1^−/−^ versus control mice (*p = *0.03, Fig. 5C, 5E). No such differences in energy expenditure or physical activity were observed between MIC-1^−/−^ and MIC-1^+/+^ male mice (Fig. 4B, 4C, 4D, 4E).

**Figure 6:**
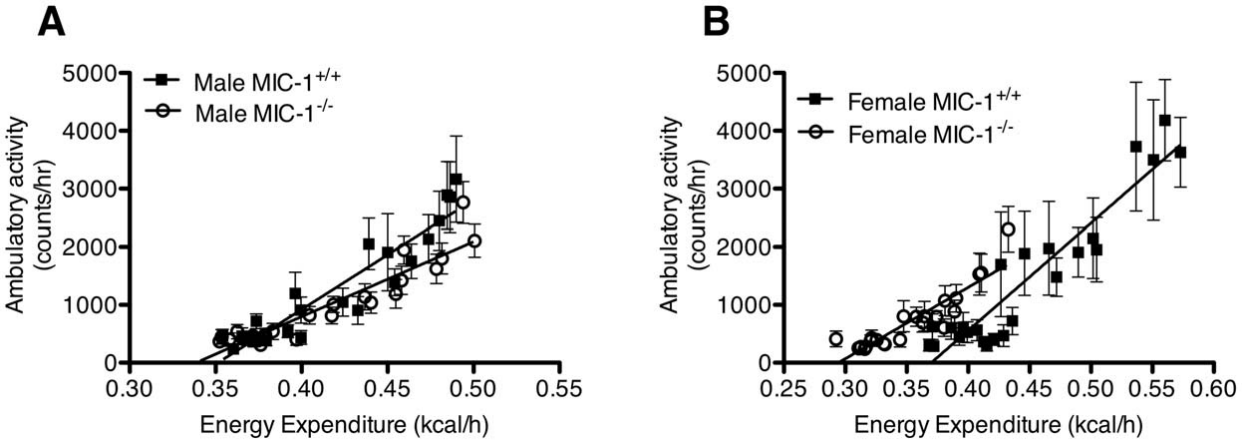
Major contribution to genotypic difference in total EE was basal metabolism. Correlation between physical activity and EE was based on average values collected over 24 h. Each point represents data collected in 1-h intervals from the (A) male MIC-1−/− and control mice (Trend line equation: MIC-1^−/−^ *y* = 12932*x* –4375 *R^2^* = 0.8705, control *y* = 18893*x* –6637 *R^2^* = 0.8813) and (B) female MIC-1^−/−^ and control mice (Trend line equation: MIC-1^−/−^ *y* = 18517*x* –6851 *R^2^* = 0.8796, control *y* = 12326*x* –3628 *R^2^* = 0.8261). Basal metabolic rate is determined using the function from the trend line and extrapolating to set the physical activity to zero. No significant difference in basal metabolic rate between the male genotypes (0.35±0.01 *vs* 0.34±0.02, respectively, *p* = 0.23, *n = *15/group). Basal metabolic rate was significantly lower in the female MIC-1^−/−^ mice compared to control (0.37±0.02 vs 0.29±0.01, respectively, *p*<0.01, *n = *9/group). Data are means ± SE.

To determine the likely contribution of changes in physical activity to changes in energy expenditure, correlation analysis was performed using hourly data from individual mice. There was a positive correlation between energy expenditure and physical activity within all the groups (*p<*0.02 by Pearson correlation for all groups, Fig. 6A and 6B). In both males and females, the difference in the slope of the regression line is significantly different for MIC-1^−/−^ and MIC-1^+/+^ mice (*p<*0.01 in all group, Fig. 6), indicating that the energy cost of activity was different between genotypes. Further, to estimate basal metabolic rate, the function from the trend line was used to extrapolate physical activity to zero, with the point at which the line crosses the X-axis signifying basal metabolic rate. While there was no genotypic difference in basal metabolic rate between males (Fig. 6A), female MIC-1^−/−^ displayed a significantly lower basal metabolic rate compared to wild type controls (*p<*0.01) (Fig. 6B). These data indicate that there is a fundamental metabolic difference between the male and female MIC-1^−/−^ mice relatively to their matched controls, suggesting MIC-1/GDF15 exerts its effects differentially on male and female mice. The difference in total energy expenditure between the female genotypic groups may have been more affected by changes in basal metabolic rate and less by the physical activity. Both of these are likely to contribute to the body weight difference displayed in female MIC-1^−/−^ versus control mice.

### MIC-1/GDF15 Reduces Food Intake and Induces Weight Loss in MIC-1^−/−^ and MIC-1^+/+^ Mice

**Figure 7:**
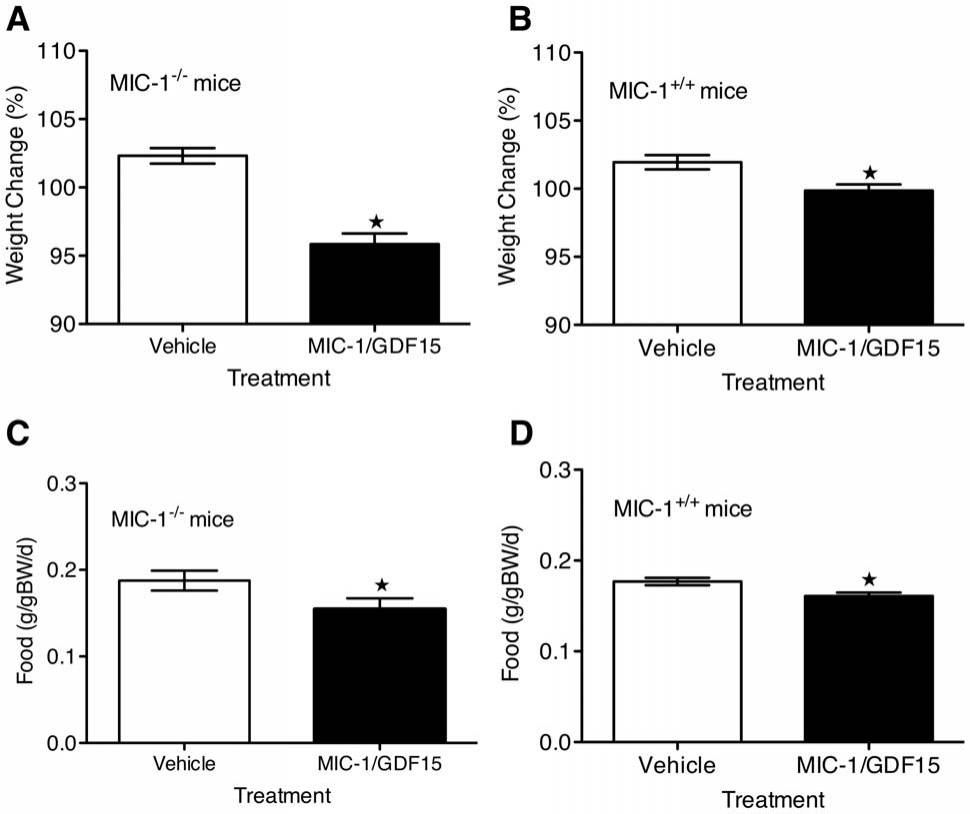
Physiological levels of human MIC-1/GDF15 reduce weight and food intake in mice. Male MIC-1^−/−^ and MIC-1^+/+^ mice were infused with human MIC-1/GDF15 (1ug/20gBW/d) or vehicle via osmotic mini-pump. Food intake, body weight and serum levels of human MIC-1/GDF15 were measured on day 5 of infusion. (A) MIC-1/GDF15-treated MIC-1^−/−^ mice had an average serum MIC-1/GDF15 level of 643±67 pg/ml and weighed 95.86±0.77% of their starting body weight whilst vehicle-treated mice weighed 102.3±0.75% of their starting weight (*n* = 6/group, *p*<0.01 *unpaired t-test*). (B) MIC-1/GDF15-treated MIC-1^+/+^ mice had an average serum MIC-1/GDF15 level of 576±45 pg/ml and weighed 99.86±0.47% of their starting weight whilst vehicle-treated mice weighed 102±0.52% (*n* = 14, *p* = 0.01 *unpaired t-test*). This decreased body weight in both genotypes was associated with reduced food intake. (C) MIC-1/GDF15-treated MIC-1^−/−^ and (D) MIC-1/GDF15-treated MIC-1^+/+^ consumed significantly less food than the matched vehicle-treated mice of same genotype (MIC-1^−/−^ *n* = 6/group, *p* = 0.04; MIC-1^+/+^ *n* = 14/group, *p*<0.01 *unpaired t-test*). Data expressed as mean ± SE.

To investigate whether the increased body weight of MIC-1^−/−^ mice was specifically due to germline gene deletion of MIC-1/GDF15 and not resulting from any unrecognized compensatory or developmental changes in MIC-1^−/−^ mice, we continuously infused male MIC-1^−/−^ and MIC^+/+^ mice with MIC-1/GDF15 (1–2 µg/d) via osmotic minipumps. Infusion of MIC-1/GDF15 over 5 days resulted in increased circulating human MIC-1/GDF15 levels from zero to 643±67 pg/ml and 576±45 pg/ml in MIC-1^−/−^ and MIC-1^+/+^ mice, respectively. As discussed below, this would have the effect of increasing total MIC-1/GDF15 levels in MIC-1^−/−^ mice to about the middle of the human normal range and in MIC-1^+/+^ mice to the top of the human normal range. This infusion of MIC-1/GDF15 resulted in reduced body weight gain relative to syngeneic vehicle-infused controls (Fig. 7A and 7B, MIC-1^−/−^ *p*<0.01; MIC-1^+/+^ *p* = 0.01), coupled with a significant reduction in food intake (Fig. 7C and 7D, MIC-1^−/−^ *p* = 0.04; MIC-1^+/+^ *p*<0.01).

## Discussion

In addition to high circulating levels of MIC-1/GDF15 mediating anorexia/cachexia in disease states [9], this study demonstrates that changes in MIC-1/GDF15 in the physiological range modifies feeding behavior and body weight in mice. The physiological range of MIC-1/GDF15 in mouse blood is currently unknown due to the lack of any immunoassay for, or monoclonal antibody to murine MIC-1/GDF15. Taken that the normal range for MIC-1/GDF15 in human serum is 150–1150 pg/ml [8] and assuming MIC-1/GDF15 serum levels are similar in humans and in mice, this means that the level of human MIC-1/GDF15 introduced in MIC-1^−/−^ and MIC-1^+/+^ mice was at middle or the upper limit of the normal human physiological range, respectively. Since this resulted in decreased body weight and food intake in both groups relatively to its control, it indicates that receptor upregulation or developmental changes in MIC-1^−/−^ mice are not responsible for human MIC-1/GDF15-induced changes in food intake and body weight, suggesting that there is a specific physiological role of MIC-1/GDF in regulation of energy intake, storage and expenditure.

Although there were distinct differences between male and female mice that are discussed below, in general MIC-1/GDF15 deficient mice exhibited increased body weight, adiposity and – in female mice – food intake. This phenotype was associated with a decrease in physical activity and basal metabolic energy expenditure in female animals. These changes in food intake and body weight in male and female mice were due to lack of serum MIC-1/GDF15 in the knockout animals, since administration of physiologically relevant amounts of human MIC-1/GDF15 decreased food intake and body weight in both MIC-1^−/−^ and syngeneic MIC-1^+/+^ mice.

Despite having a similar phenotype with respect to increased body weight and adiposity, the effects of MIC-1/GDF15 gene deletion was greater in female than in male mice and the underlying physical/metabolic changes differed between the sexes in some aspects. This suggests that MIC-1/GDF15 exert its effect differentially between male and female animals. This is consistent with epidemiological data from human cohorts, where there are sex-related differences in the relationship between MIC-1/GDF15 and anthropometric measurements (e.g. waist-to-hip ratio) [25], [26].

In mice, female but not male MIC-1^−/−^ mice displayed a significant reduction in lean mass, a significant increase in spontaneous food intake as well as significantly reduced energy expenditure, basal metabolic rate and physical activity compared to control mice. Although white adipose tissue consumes/stores energy and helps to regulate metabolic rate, lean mass consumes much more energy than the fat mass [27], [28]. Therefore, the relatively reduced lean mass seen only in the female MIC-1^−/−^ female mice may have contributed to the associated reduction in energy expenditure and basal metabolic rate in these animals, and may help to explain the greater difference in body weight of the female MIC-1^−/−^ versus control mice.

Whilst male mice MIC-1^−/−^ weight more, and are more obese than their syngeic controls, this difference is less than in females and its aetiology is less clear. The increase in spontaneous food intake in male MIC-1^−/−^ mice was not statistically significant, either because no real difference existed or because the study was underpowered to detect a small difference. However, it is noteworthy that in humans, sustained small changes in daily energy intake, as low as 10 kcal, are capable of altering body weight and composition [29], [30]. Additionally, it is not uncommon for C57BL5 mice to display changes in body weight and body composition with little or no changes in energy intake [31].

Like MIC-1/GDF15, various other members of the TGF-b superfamily are also involved in the regulation of adipogenesis and energy metabolism by signaling through the smad and p38 mitogen-activated protein kinase (p38MAPK) signaling pathways, or alternatively signaling through other members of MAPK family such as ERK and JNK [32]. Whilst the effects are controversial, in general, bone morphogenetic proteins (BMPs) direct commitment of preadipocytes to either the white (BMP2 and BMP4) or the brown adipocyte (BMP7) lineage. [BMP7 also increases mitochondrial biogenesis leading to increased energy expenditure [27], [33]–[36]. By contrast, TGF-b1 and activin inhibit the differentiation of preadipocytes to mature adipocytes and hamper lipid accumulation [37]–[39]. Additionally, in mouse knockout models, growth differentiation factor 3 (GDF3) deficient mice displayed increased basal metabolic rate, and myostatin deficient mice displayed decreased skeletal muscle mass with increased adiposity [40]–[42], which contrasted to what female MIC-1^−/−^ exhibited, suggesting a novel role of MIC-1/GDF15 in the regulation of metabolic rate and possibly also myogenesis. Both male and female MIC-1^−/−^ mice have increased white but no alteration in brown adipose tissue mass. This could indicate a possible role for MIC-1/GDF15 in inhibition of lipid formation and/or accumulation. However the increase in adiposity MIC-1^−/−^ mice is more likely to have developed because of increase in food intake, at least in female mice. Whilst in male mice, we could not identify significant alteration in food intake, small differences, significant enough to alter body weight and compositions could still exist but are beyond our capacity to detect in this study.

MIC-1/GDF15 was first identified as an appetite regulator when it was discovered that its overexpression in cancer and other diseases lead to anorexia/cachexia. The data in this study indicates that under physiological conditions, MIC-1/GDF15 also plays a small role in the regulation of energy intake and expenditure, with greater effects in females than in males. Thus it would appear that diseases associated with marked increases in MIC-1/GDF15 expression subvert a normal physiological pathway to cause anorexia/cachexia. A better understanding of this pathway is important for a complete understanding of energy homeostasis and more effective therapy of the anorexia/cachexia syndrome.
